# Exploratory assessment of preconception phthalate exposure on pregnancy and offspring health outcomes in mice

**DOI:** 10.1210/jendso/bvag010

**Published:** 2026-01-23

**Authors:** Maryam Afghah, Ansley C Elkins, Paige C Powell, Mary E Mulligan, Mary C Boland, Alexandra P Suggs, Melissa A Walker, Grace E Brenner, Lindsay C Johnson, Zachary J Padgett, Kylie D Rock

**Affiliations:** Department of Biological Sciences, Clemson University, Clemson, SC 29634, USA; Department of Biological Sciences, Clemson University, Clemson, SC 29634, USA; Department of Biological Sciences, Clemson University, Clemson, SC 29634, USA; Department of Biological Sciences, Clemson University, Clemson, SC 29634, USA; Department of Biological Sciences, Clemson University, Clemson, SC 29634, USA; Department of Biological Sciences, Clemson University, Clemson, SC 29634, USA; Department of Biological Sciences, Clemson University, Clemson, SC 29634, USA; Department of Biological Sciences, Clemson University, Clemson, SC 29634, USA; Department of Biological Sciences, Clemson University, Clemson, SC 29634, USA; Department of Biological Sciences, Clemson University, Clemson, SC 29634, USA; Department of Biological Sciences, Clemson University, Clemson, SC 29634, USA; Center for Human Genetics, Clemson University, Greenwood, SC 29646, USA

**Keywords:** phthalates, endocrine-disrupting chemicals, preconception, placenta, developmental programing

## Abstract

Understanding how endocrine-disrupting chemicals influence reproductive success requires attention to sensitive windows beyond gestation, including the understudied preconception period. In this exploratory pilot study, female CD-1 mice were exposed to a human-relevant phthalate mixture (200 µg/kg/day) for 30 days prior to mating. Although implantation and litter size were unaffected, exposed dams exhibited nonsignificant shifts in estrus cyclicity, spending more time in proestrus and less in metestrus. Maternal liver transcriptomics revealed persistent changes more than a month after exposure ceased, with differential expression of genes involved in mitochondrial metabolism, oxidative phosphorylation, and xenobiotic processing, suggesting long-term metabolic reprograming in the absence of overt toxicity. Maternal effects coincided with developmental alterations at mid-gestation. At E14.5, fetuses from exposed dams were heavier, and placentas displayed expansion of the junctional zone, a region critical for endocrine function. This early growth enhancement reversed later in life, as exposed male offspring exhibited reduced adult body weight, consistent with altered developmental programing. Transcriptomic profiling revealed pronounced sex-specific placental responses: female placentas exhibited extensive reprograming across immune, metabolic, and extracellular matrix pathways (518 differentially expressed genes [DEGs]), whereas male placentas showed minimal differential expression (9 DEGs), despite enrichment for RNA processing and mitochondrial pathways. Adult offspring livers also displayed sex-specific transcriptional signatures, with exposed females downregulating metabolic and immune-regulatory genes and exposed males upregulating inflammatory pathways. Collectively, these hypothesis-generating findings provide early evidence that preconception exposures can shape maternal physiology, placental development, and long-term offspring health, highlighting the preconception period as a critical yet understudied window of susceptibility.

Reproductive health challenges, including difficulties conceiving, maintaining a healthy pregnancy, or supporting optimal fetal development, affect a substantial proportion of the global population and carry meaningful public health implications (1-3). Although these outcomes arise from diverse anatomical, genetic, and endocrine influences, a growing body of evidence indicates that environmental chemical exposures contribute to reproductive dysfunction more broadly (4-11). Endocrine-disrupting chemicals (EDCs), in particular, have received increased attention for their ability to interfere with hormonal signaling during critical windows of reproductive development and function, raising concern about their impact on both parental health and offspring outcomes (12-19).

More than 85,000 synthetic chemicals are registered for commercial use in the United States, and over 1400 have been identified as EDCs based on their ability to disrupt endogenous hormone activity (20, 21). These chemicals are pervasive in modern life, found in plastics, personal care products, food packaging, pesticides, flame retardants, and household goods (22, 23). While some EDCs have been banned or regulated, many remain in circulation, and humans are routinely exposed to complex mixtures of low-dose chemicals throughout their lifetimes (22, 24). Epidemiological studies have associated EDC exposure with menstrual irregularities, anovulation, recurrent pregnancy loss, and adverse birth outcomes (25-28). However, establishing causality and identifying the precise biological mechanisms by which these exposures impair conception and impact pregnancy outcomes has been hindered by the complexity of real-world exposures, ethical constraints in human studies, and limitations of traditional toxicology models that often focus on single compounds at high doses.

Phthalates are among the most ubiquitous and extensively studied EDCs with known effects on female reproductive health. These multifunctional chemicals are used as plasticizers in polyvinyl chloride (PVC) products, as well as solvents and fragrance carriers in cosmetics, lotions, perfumes, and other personal care items (29-32). Due to their non-covalent incorporation into consumer goods, phthalates readily leach into the environment, leading to chronic, low-level exposure via ingestion, inhalation, and dermal absorption (33). Biomonitoring studies have consistently detected phthalate metabolites in human urine, blood, and even follicular fluid, with women often showing higher body burdens than men, likely due to gendered differences in product use (34). Experimental research has demonstrated that phthalate exposure during pregnancy can impair ovarian steroidogenesis, alter uterine gene expression, reduce endometrial receptivity, and contribute to adverse offspring health outcomes, including reproductive and metabolic dysfunction (34-43). However, little is known about preconception exposure or the mechanisms through which it affects fertility, fecundity, and pregnancy outcomes.

The preconception period represents a particularly vulnerable window during which EDCs can disrupt key physiological processes required for successful reproduction. Beginning at puberty, female reproductive hormones undergo tightly regulated cyclical fluctuations that orchestrate ovulation and prepare the uterus for implantation (44, 45). While this hormone-driven process is intentionally disrupted by contraceptives to prevent pregnancy, widespread exposure to environmental EDCs may unintentionally interfere with the same endocrine pathways (12, 46-49). These exposures are continuous, low-dose, and largely unavoidable, making their effects on reproductive readiness especially concerning. Perturbations to hormonal signaling during the preconception window, defined here as the period between puberty and pregnancy, could impair oocyte maturation, ovulation, uterine receptivity, and implantation. Although a growing number of epidemiological studies have reported associations between EDC exposure and reduced fertility, establishing causality and elucidating mechanisms remains challenging (50-53). To overcome these barriers, the current pilot study leverages a murine model to investigate how preconception exposure to an environmentally relevant phthalate mixture affects maternal reproductive physiology, implantation success, placenta form and function, and offspring health outcomes. We hypothesize that preconception phthalate exposure will disrupt hormone dependent processes including the estrus cycle and implantation, leading to downstream consequences for placental architecture and function and offspring health. By leveraging a physiologically relevant exposure paradigm and focusing on an etiologically important window of reproductive vulnerability, this work aims to elucidate the causal links between EDCs and reproductive success, with implications for understanding infertility, pregnancy loss, and intergenerational health effects in exposed populations.

## Materials and methods

### Animals

All animal care, maintenance, and experimental procedures adhered to the standards outlined by the Animal Welfare Act and the US Department of Health and Human Services Guide for the Care and Use of Laboratory Animals and were approved by the Clemson University Institutional Animal Care and Use Committee (IACUC). The Animal Research: Reporting of *In Vivo* Experiments (ARRIVE) Guidelines Checklist for Reporting Animal Research was used in construction of this manuscript with all elements met (54). A supervising veterinarian oversaw and monitored all procedures throughout the duration of the study. For each aim, female and male CD-1 mice were obtained from Charles River Laboratories (Raleigh, North Carolina) and housed at the Godley-Snell Research Center at Clemson University, an AAALAC-accredited biological resource facility. Animals were maintained under standard laboratory conditions with a controlled temperature of 25 °C, 12:12 hour light–dark cycle, and average humidity between 45% and 60%. Mice were allowed a 2-week acclimation period prior to initiation of experimental procedures. Housing and husbandry practices were specifically designed to minimize unintended exposure to EDCs. All mice were provided a soy-free diet (Teklad 2020), housed in poly-sulfone cages, supplied filtered drinking water via glass water bottles fitted with metal sippers, and bedded with woodchip material, in accordance with best practice guidelines for endocrine disruptor research (16, 17, 55-58).

### Dosing prep

Oral exposure to a phthalate mixture dissolved in a tocopherol-stripped corn oil vehicle was used as the route of administration for all animals. To prepare the concentrated stock solution, individual phthalates were pipetted directly into a pre-weighed vial placed on an analytical balance. The volume of each chemical added was recorded to precisely achieve the target mass, based on the relative percentage composition calculated from previous assessments of human exposure (Table 1) (59-61).

**Table 1:** Phthalates used in mixture, percentage of each phthalate in mixture, and calculated daily exposure in mice with relevance to human daily exposures

Phthalate	Abbreviation	% of dosing mixture	Mouse exposure (μg/kg/day)*^a^*	Human exposure (μg/kg/day) (59, 60)
Diisobutyl phthalate	DiBP	8	1.6-16	0.12-1.4
Diisononyl phthalate	DiNP	15	3-30	≤26
Di(2-ethylhexyl) phthalate	DEHP	21	4.2-42	3-30
Benzylbutyl phthalate	BzBP	5	1-10	0.26-0.88
Diethyl phthalate	DEP	35	7-70	2.3-12
Dibutyl phthalate	DBP	15	3-30	0.84-5.22

^*a*^Mouse exposure based on the selected 200 μg/kg/day dose.

**Figure 1:**
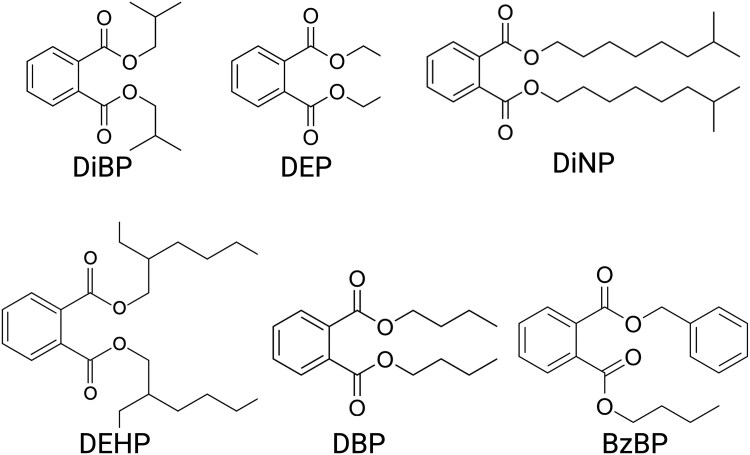
Chemical structures of the six phthalates that make up the phthalate mixture in this study. DiBP, diisobutyl phthalate; DiNP, diisononyl phthalate; DEHP, di(2-ethylhexyl) phthalate; BzBP, benzylbutyl phthalate; DEP, diethyl phthalate; DBP, dibutyl phthalate. Created with BioRender.com

The final mixture included benzylbutyl phthalate (BzBP; CAS No. 85-68-7), diisobutyl phthalate (DiBP; CAS No. 84-69-5), diisononyl phthalate (DiNP; CAS No. 28553-12-0), dibutyl phthalate (DBP; CAS No. 84-74-2), di(2-ethylhexyl) phthalate (DEHP; CAS No. 28553-12-0), and diethyl phthalate (DEP; CAS No. 84-66-2). Phthalates were obtained from Sigma-Aldrich (purity: ≥98%). Tocopherol-stripped corn oil (MP Biomedicals) served as the vehicle control for all dosing groups (Fig. 1). The proportional composition of the mixture was based on urinary phthalate metabolite concentrations measured in pregnant women enrolled in the Children's Environmental Health Research Center at the University of Illinois (59-61). Metabolite concentrations were used to recalculate the proportions of diester phthalates representing typical human exposures (Table 1). Working dosing solutions were prepared to achieve the targeted exposure level of 200 μg/kg/day by serial dilution of the concentrated stock in corn oil. All dosing solutions were mixed overnight on an orbital shaker and stored in amber glass vials to prevent photodegradation until use.

### Animal husbandry and exposure

Adult CD-1 mice (8 females and 4 males) were obtained from Charles River Laboratories (Raleigh, NC). Breeding pairs were established by housing two females with a single male to generate a colony of in-house offspring for exposure. To ensure uniformity in pubertal development at the start of exposure, female offspring were weaned at PND 21 and randomly selected for experimental use at postnatal day (PND) 30. All animals were weighed daily throughout the exposure period, and estrus cyclicity was tracked by daily vaginal smear (∼10:00 Am) using sterile physiological saline (0.9% NaCl) as described previously (62). Lavage samples were stained using 0.05% toluidine blue to identify estrus cycle stages, serving as noninvasive biomarkers of endocrine and reproductive function (Fig. S1) (63).

**Figure 2:**
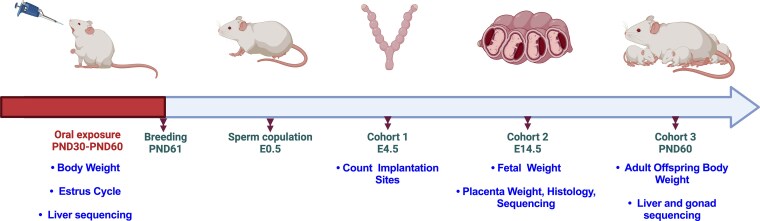
Timeline of experimental design. Pregnant females were split into three cohorts to evaluate impacts on: (1) implantation (E4.5), (2) fetal and placental development (E14.5), and (3) adult offspring health outcomes (PND 60). Maternal body weight and estrus cycle were tracked throughout the duration of exposure. Implantation sites were counted at E4.5. Litter size was counted, and fetal and placental weights were collected at E14.5. Adult offspring bodyweight and estrus cycle were tracked between PND 30 and 60. Finally, tissues including livers, placentas were collected for RNA-Sequencing. Created with BioRender.com

Beginning on PND 30, female mice were randomly assigned to receive either the 200 μg/kg/day phthalate-mixture (n = 25) or vehicle control (n = 20) via daily oral pipetting for 30 consecutive days, concluding at PND 60. All doses were given in 21 to 25 μL volumes based on their body weight (Supplemental Data 1, Table S1) (63). Following the final day of dosing at PND 61, 2 virgin female mice were paired with a single non-littermate male to maintain outbred genetic heterogeneity. Upon detection of a vaginal sperm plug, males were removed, and dams were singly housed. The morning a sperm plug was detected was considered embryonic day (E) 0.5. Pregnant dams were randomly assigned to one of three cohorts defined by their experimental endpoint, cohort 1 to assess implantation, cohort 2 to assess placental structure and function, and cohort 3 to evaluate long-term offspring health outcomes (Fig. 2).

### Tissue collection

Animals were sacrificed using a method approved by the Panel on Euthanasia of the American Veterinary Medical Association. For cohort 1, mice were fully anesthetized with isoflurane inhalant prior to tail vein dye injection. Cohorts 2 and 3 were euthanized using a standardized CO₂ inhalation protocol in accordance with AVMA Guidelines for the Euthanasia of Animals. CO₂ exposure was selected because it allows rapid and consistent loss of consciousness, minimizes handling stress, and is compatible with downstream histological and transcriptomic endpoints. Importantly, all dams, control and phthalate-exposed, underwent the identical CO₂ procedure, ensuring that any nonspecific transcriptional effects induced by brief hypoxia would be equally distributed across groups. Tissue collection (placenta, fetal liver, and fetal gonad) occurred immediately after euthanasia to minimize the window during which CO₂-associated gene expression changes can occur. Prior studies indicate that euthanasia methods, including pentobarbital, isoflurane, propofol and CO₂ can induce transcriptional alterations with the strongest effects observed in neural tissues but minimal effects reported in peripheral or extraembryonic tissues within short exposure intervals (64-67). Furthermore, comparative studies highlight that CO₂ has less of an impact on gene and protein expression/activity compared to drug based euthanasia methods. Thus, the CO_2_ approach utilized in this study is unlikely to differentially influence placenta transcriptional endpoints.

**Figure 3:**
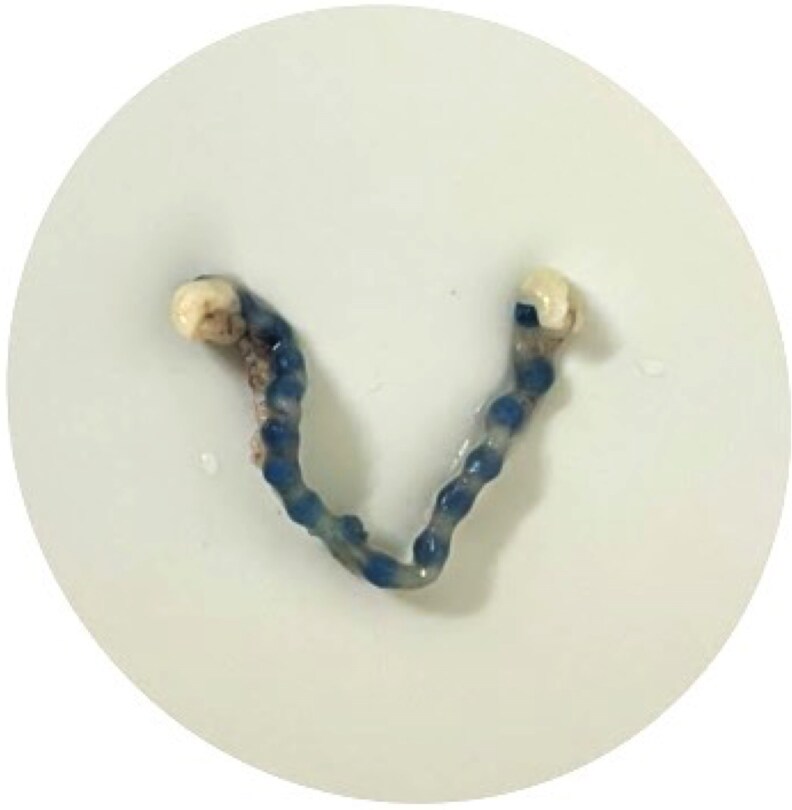
Representative image of Chicago blue–stained implantation sites.

*Cohort 1:* Implantation success was assessed on E4.5 by intravenous tail vein injection of 1% Chicago Blue dye (Sigma-Aldrich) in sterile saline for dams exposed to 200 μg/kg/day phthalate-mixture (n = 4) or corn oil (n = 3). Uteri were subsequently examined for dye localization marking implantation sites (Fig. 3).

*Cohort 2:* Fetuses and placentas were collected at E14.5 from 200 μg/kg/day phthalate-mixture (n = 5) and control dams (n = 4). Tissues were weighed immediately after collection. A small portion of tail tissue was collected from each fetus for sex determination by genotyping using primers specific to Jarid1 (5′-TGAAGCTTTTGGCTTTGAG-3′ and 5′-CCGCTGCCAAATTCTTTGG-3′) as described elsewhere (68). Each placenta was bisected, with one half flash-frozen for RNA sequencing and stored at −80 °C, and the other half fixed in 10% neutral-buffered formalin (NBF) for histopathological analysis. Based on the results of sex genotyping, one male and one female from each litter were selected for downstream analyses.

*Cohort 3:* At weaning, PND 100 dams were sacrificed, and livers were collected and flash-frozen for downstream analysis (200 μg/kg/day n = 3, control n = 3). Adult offspring livers and gonads were collected on PND 60 for both sexes and flash-frozen for downstream analysis (200 μg/kg/day n = 3, control n = 3). Postnatal weight change for two adult offspring per sex following maternal exposure was monitored from PND30 to 60 weekly (200 μg/kg/day n = 15 per sex, control n = 9 male and n = 11 female). Females born in this group were monitored daily to assess estrus cycle from PND 30 to 45 as well.

### Histology

For gross histopathological assessment, a half of a placenta, 1 per sex per litter, was initially fixed in 10% NBF. Tissues were then sequentially transferred to NBF containing 30% sucrose, followed by immersion in a solution of 30% sucrose in phosphate-buffered saline (PBS) for cryoprotection. Fixed placentas were stored at −80 °C until histological processing. Prior to paraffin embedding, tissues underwent a reverse sucrose gradient protocol, 20% for 24 hours, 10% for 24 hours, and PBS for 24 hours, to remove sucrose prior to paraffin processing. Placentas from control dams (n = 4/sex) and 200 μg/kg/day phthalate mixture group (n = 5/sex) were sent to the Clemson Light Imaging Facility (CLIF) for hematoxylin and eosin (H&E) staining and imaging. Tissues were embedded in paraffin using the Leica Histocore Arcadia Embedding Station, sectioned at 5 μm thickness with the Leica Histocore Biocut Rotary Microtome, and mounted on glass slides. Sections were de-waxed and rehydrated using a series of xylene and ethanol baths, where ethanol concentrations progressively decreased from 100%-50% followed by a final rinse in PBS. Slides were stained with hematoxylin and eosin (H&E) for morphological analysis using an H&E staining kit (Abcam) according to the manufacturers protocol. Post-processing and staining, two female placentas from the control group were excluded from the study due to compromised tissue integrity, resulting in a final sample size of n = 2 female control placentas. Because this sample size is insufficient for within-sex or between-sex hypothesis testing, placental histology analyses were performed either in the combined dataset (male + female) or in males only, where adequate replication existed. Due to limited female control samples (n = 2), female-only analyses were not conducted, and sex differences could not be formally tested in this histological endpoint. Images were taken using a DSX1000 Digital Microscope (Olympus). Area measurements for the whole placenta and specific placental zones (labyrinth, junctional and decidua) were determined using ImageJ (version1.54g). Areas were measured by two blinded researchers and the average area from those two was used for data analysis.

### RNA-Sequencing

RNA-seq as an initial hypothesis-generating approach was performed on E 14.5 placentas, PND 60 offspring liver and gonads, and PND 100 dam livers. For placental RNA-seq, we selected one male and one female placenta per litter based on RNA integrity (RIN), tissue quality, and preservation of all major placental compartments following dissection (Supplemental Data 1, Table S2) (63) When multiple conceptuses of the same sex within a litter met these criteria, we selected the sample with the highest RIN value to ensure optimal sequencing quality. Because intrauterine endocrine signaling can be influenced by the sex of adjacent fetuses, we additionally prioritized placentas flanked by mixed-sex neighbors (one male and one female) when possible (69, 70). This approach reduced local sex-biased paracrine variability while maintaining consistent sample quality across litters (70). Uterine position metadata (left/right horn and relative position within the horn) were recorded for all conceptuses; however, uterine position itself (eg, left, right, top, bottom) was not used as a selection criterion due to limited evidence for strong positional effects on placental morphology or growth in standard mouse pregnancies.

Because the placenta was our primary focus with relevance to pregnancy outcomes, offspring liver and gonadal tissues and maternal liver samples were not initially collected during necropsy for cohort 3, which limited our sample numbers for sequencing. However, we used this constraint as an opportunity to generate preliminary, hypothesis-generating transcriptomic data from the specimens that were available, providing early insight into potential tissue-specific responses that can inform the design of future, fully powered studies.

Approximately 30 mg of tissue were used for RNA extraction with the Qiagen RNEasy Miniprep kit according to the manufacturer protocol (Qiagen, Cat. 74134). Total RNA was submitted to Novogene (Sacramento, CA, USA) for mRNA sequencing using the Illumina platform. RNA quality was determined using an Agilent 5400 Bioanalyzer and only samples that passed Novogene quality control criteria were used for sequencing. Placenta (control n = 4/sex and 200 μg/kg/day phthalate mixture n = 5/sex), offspring liver (control female n = 3, control male n = 2, 200 μg/kg/day phthalate mixture n = 3/sex), offspring gonad (control n = 3/sex and 200 μg/kg/day phthalate mixture n = 3/sex), and dam liver (control female n = 3, 200 μg/kg/day phthalate mixture exposed female n = 3) mRNA was isolated from total RNA using poly-T oligo-attached magnetic beads, followed by heat fragmentation and first-strand cDNA synthesis using random hexamer primers. Second-strand synthesis was performed using dUTP to maintain strand specificity (71). Libraries underwent end repair, A-tailing, adapter ligation, size selection, PCR amplification, and final purification. Library quality and concentration were assessed using Qubit fluorometry, real-time PCR, and an Agilent 5400 Bioanalyzer. Quantified libraries were pooled and sequenced on Illumina platforms, according to effective library concentration and data amount.

### RNA-seq data processing

#### Data quality control

Raw data (raw reads) of fastq format were firstly processed through in-house perl scripts. In this step, clean data (clean reads) were obtained by removing reads containing adapter, reads containing ploy-N and low-quality reads from raw data. At the same time, Q20, Q30 and GC content of the clean data were calculated. All the downstream analyses were based on the clean data with high quality. The transcriptome data discussed in this publication has been deposited in NCBI's Gene Expression Omnibus (PRJNA1308639) (72).

#### Read mapping to the reference genome

The RNA-seq reads were aligned to the Mus musculus GRCm39 (mm39) reference genome (NCBI accession GCF_000001635.27). We used the NCBI RefSeq annotation, Annotation Release 109 for transcript quantification. Reference genome and gene model annotation files were downloaded from genome website directly. Index of the reference genome was built using Hisat2 v2.0.5 and paired-end clean 1 reads were aligned to the reference genome using Hisat2 v2.0.5. We selected Hisat2 (73) as the mapping tool for that Hisat2 can generate a database of splice junctions based on the gene model annotation file and thus a better mapping result than other non-splice mapping tools.

#### Quantification of gene expression level

Feature Counts (74) v1.5.0-p3 was used to count the read numbers mapped to each gene. Average uniquely mapped reads in placentas, livers, and gonads ranged between 37 and 38 million per library. And then FPKM of each gene was calculated based on the length of the gene and read counts mapped to this gene. FPKM, expected number of Fragments Per Kilobase of transcript sequence per Millions base pairs sequenced, considers the effect of sequencing depth and gene length for the read counts at the same time, and is currently the most used method for estimating gene expression levels.

#### Differential expression analysis

Differential gene expression analysis was performed using the *DESeq2 R* package (v1.20.0) (75, 76). *DESeq2* implements statistical routines for digital gene expression data based on a negative binomial generalized linear model and estimates dispersion parameters appropriate for small-n RNA-seq experiments. *P*-values were adjusted using the Benjamini–Hochberg procedure to control the false discovery rate (FDR), and transcripts with an adjusted *P*-value (*P*-adj) ≤ .05 were considered differentially expressed.

Given the limited biological replicates available in this pilot study, we applied additional filtering to increase the robustness of the results. In accordance with previously published protocols, adult offspring liver datasets were filtered to remove low-abundance transcripts detected in fewer than two samples per group (77). After filtering, a total of 14 896 genes remained for differential expression testing in males and 14 078 genes in females. Because the analysis is based on small sample sizes (eg, two biological replicates for control male adult liver), results are interpreted as exploratory and hypothesis-generating rather than confirmatory.

### Statistical methods

Statistical analysis was performed using GraphPad Prism version 10 (version 10.4.2, La Jolla, California) and the R statistical environment (version 4.4.3, R core team 2021) with statistical significance set at *alpha* = .05. Results are presented as mean ± SEM or min to max. For comparisons between two groups, unpaired Student's *t*-tests were used. Two-way ANOVA followed by LSD post hoc tests was applied for multiple group comparisons. For t-test's and ANOVA's effect size was determined by calculating an Eta squared (η^2^), effects of which are defined as small at 0.01, medium at 0.06, and large at 0.14 (78).

For the placental area analyses, we first evaluated whether litter-level clustering needed to be incorporated into the model structure. Because placentas within a litter share the same maternal physiology, uterine environment, and gestational exposures, they are not statistically independent. To quantify the contribution of this intra-litter correlation, we fit a linear mixed-effects model of log-transformed placental area with litter specified as a random intercept and compared it to a reduced model lacking this term using a restricted maximum likelihood (REML)–based random-effects ratio test. This test allowed us to determine whether including the random litter effect significantly improved model fit for placental area. As expected for placental endpoints, litter accounted for a substantial proportion of variance, supporting its inclusion in the final mixed-model framework.

For models in which fixed effects (eg, dose or sex × dose interactions) were statistically significant, we performed post hoc pairwise comparisons of estimated marginal means. These contrasts were derived from the fixed-effect covariance structure while retaining the random litter term, ensuring that group-level comparisons appropriately accounted for the non-independence of placentas originating from the same pregnancy.

A detailed estrus cycle analysis was conducted for offspring but not for dams because the biological and statistical characteristics of the two datasets differed substantially. Maternal cycles were highly consistent, showed expected stage distributions, and demonstrated strong agreement among scorers, making traditional categorical staging sufficient. In contrast, offspring cycles were markedly more variable, with irregular transitions and disproportionate time spent in specific stages, necessitating a more rigorous scoring and analytical approach. To address this variability and scorer uncertainty, we applied a structured consensus method that combined stage calls from three observers using majority rule and assessed inter-rater reliability via Fleiss’ κ (79, 80).

Estrus stage calls were generated independently by three trained observers and harmonized into a single consensus stage for each animal on each day. Each dataset contained 15 daily columns with categorical stage designations (proestrus, estrus, metestrus, diestrus; P/E/M/D). For every animal × day, observer calls were merged using a majority-rule approach, in which the stage selected by at least two of three observers was assigned as the consensus stage. In the rare instance in which all three observers disagreed (ie, three unique stage calls), ties were resolved using a predetermined hierarchical phase order (P > E > M > D). For quality assessment, each day was additionally flagged to indicate whether observers showed complete agreement or any disagreement (ie, more than one unique stage call). Using the finalized consensus series for each animal, we calculated the percentage of monitored days spent in each stage. Inter-rater agreement among the three estrous-cycle scorers was fair to moderate (Fleiss’ κ = 0.36), consistent with expected variability in cytological staging, particularly on transition days (81). This level of agreement further justified the use of a consensus-based approach to harmonize daily stage calls.

For sequencing results over-representation analysis (ORA) was performed using the *enrichGO()* and *enrichKEGG()* functions on the subset of significantly differentially expressed genes (DEGs). Due to the limited number of DEGs meeting the *P*-adjusted cutoff of *P* < .05 for the dam liver, male placenta, male and female offspring liver, and male and female offspring gonads ORA was performed on DEGs with unadjusted *P* < .05. However, for female placenta we ran ORA on the list of 518 significantly DEGs, *P*-adjusted < .05. Enrichment scores were calculated as −log10(adjusted *P*-value), with significance set at adjusted *P*-values (FDR) < .05. These functions identify Gene Ontology (GO) terms and Kyoto Encyclopedia of Genes and Genomes (KEGG) pathways that are statistically over-represented, irrespective of whether they were up or downregulated among the DEGs.

In addition to ORA, we performed Gene Set Enrichment Analysis (GSEA) using the *clusterProfiler*R package and the same DEG *P* and *P*-adjusted value cutoffs as descrived for ORA. For each comparison, all detected genes were ranked by their log₂ fold change from the DESeq2 results, and this ranked gene list was used as input to *gseGO()*. GSEA was conducted against Gene Ontology (GO) terms using the *Mus musculus* annotation database (*org.Mm.eg.db*), with ontology set to “ALL” to interrogate biological process, cellular component, and molecular-function categories. Gene sets with a minimum size of 3 and a maximum of 800 genes were retained (minGSSize = 3, maxGSSize = 800). Enrichment statistics and normalized enrichment scores (NES) were calculated using the default *clusterProfiler* implementation, and pathways with nominal *P* < .05 were considered significantly enriched, consistent with the exploratory nature of this pilot study. To visualize functionally related pathways, we computed pairwise semantic similarity among enriched GO terms using pairwise_*termsim()* and plotted clustered term networks with *treeplot()*, alongside standard dot plots of the top enriched categories.

## Results

### Maternal bodyweight, estrus cycle, implantation, litter size, and liver RNA-seq

**Figure 4:**
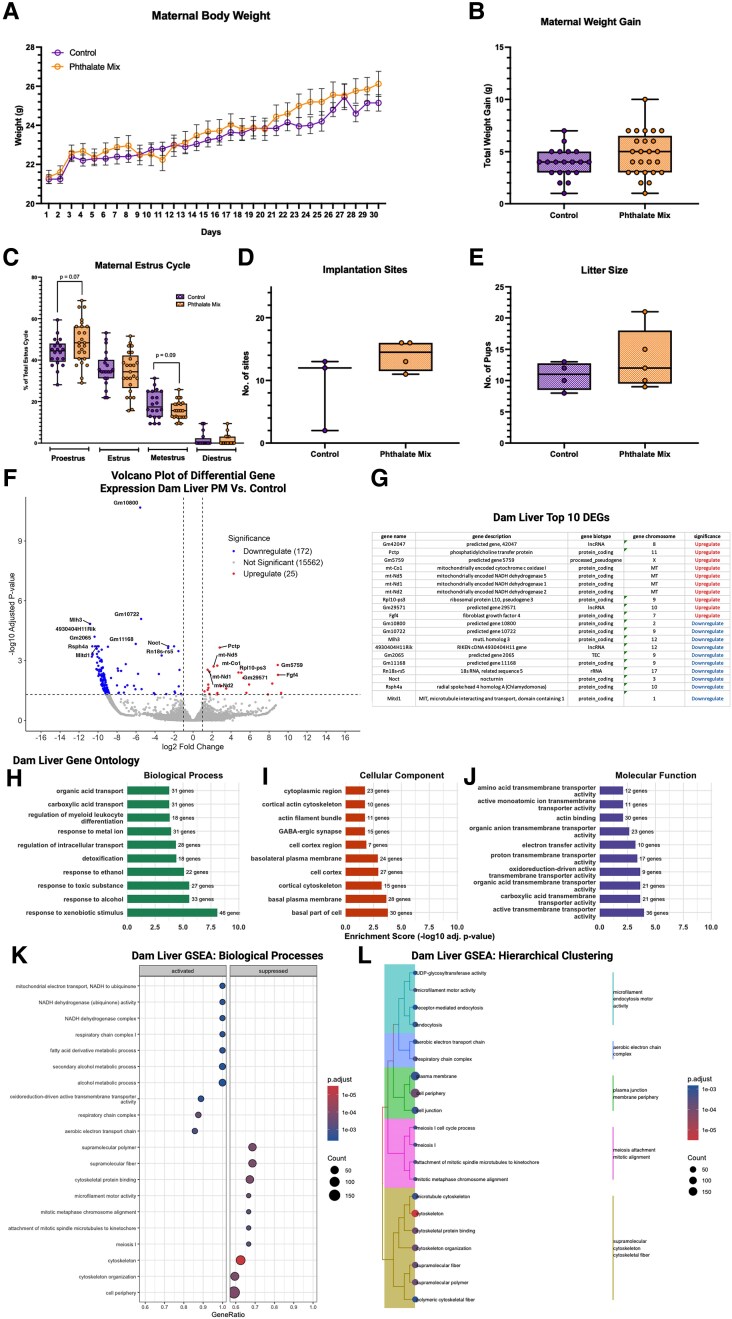
Dams exposed to phthalates during the preconception window exhibited no significant alterations in body weight or fertility, though modest disruptions in estrus cycling during exposure and persistent alternation in maternal liver gene expression post weaning were observed. Phthalate exposure had no significant impact on maternal bodyweight (A) over time or (B) total weight gain. No significant effect of exposure was observed on (C) number of days in each stage of the estrus cycle; however, t-test revealed an increased trend in the number of days in proestrus and decrease in metestrus compared to controls; n = 20 control dams; n = 25 phthalate mixture dams. Phthalate exposure didn’t significantly change the number of (D) implantation sites; n = 3 control dams; n = 4 PM dams; or (E) pups per litter; n = 4 control dams; n = 5 phthalate mixture dams. (F) Volcano plot demonstrating upregulation in 25 genes and downregulation in 172 genes following preconception exposure to phthalate mixture. (G) List of top 10 genes upregulated and downregulated, suppression of mitochondrial processes of dam's liver in PND 100 supported by (H) GO analysis on biological processes. (I) GO enrichment of cellular components. (J) GO enrichment of molecular function, validated by (K) GSEA analysis showing downregulation of mitochondrial respiration, respiratory chain complex assembly, oxidative phosphorylation, and ATP generation. (L) Hierarchical clustering of GSEA-enriched terms further demonstrated grouping suppressed pathways related to energy production, electron transport, and mitochondrial organization. Line graph depicts mean ± SEM. Boxplots depict mean, min, and max. Line graphs depict mean ± SEM.

Maternal body weight was tracked from PND 30 to 60 (Supplemental Data 1, Table S3) (63). No significant differences in weight gain over time or total weight gained were observed in exposed dams compared to controls (Fig. 4A and 4B; Supplemental Data 1, Table S4) (63). Maternal estrus cycle appeared to be impacted by phthalate exposure, with more time spent in proestrus (F (24, 19) = 2.258, *P* = .07, η^2^ = 0.07) and less time spent in metestrus (F (24, 19) = 2.283, *P* = .09, η^2^ = 0.06), but the effect did not reach statistical significance (Fig. 4C; Supplemental Data 1, Table S5) (63). Phthalate exposure also had no significant effect on the number of implantation sites at E 4.5 (Fig. 4D; Supplemental Data 1, Table S6) (63) or the number of pups per litter at E 14.5 (Fig. 4E). Given the limited sample size for this early timepoint, we view these implantation-site findings as exploratory and note that they should be interpreted with appropriate caution. Together, these data indicate that phthalate exposure did not produce statistically detectable changes in estrus cycle patterns, although some trends warrant further investigation into preconception phthalate exposure and maternal endocrine disruption.

To assess whether preconception phthalate exposure produced persistent molecular effects in dams after pregnancy and lactation, we performed RNA-seq on maternal liver tissue collected at PND 100 (post-weaning; Supplemental Data 1, Table S7) (63). Principal components analysis (PCA) and hierarchical clustering did not show consistent separation of control and exposed dam livers (Fig. S2) (63). Differential gene expression analysis revealed a modest but detectable transcriptional response (Fig. 4F). A total of 197 genes were significantly altered after FDR correction, with 172 downregulated and 25 upregulated in the phthalate-exposed group. While most DEGs exhibited small to moderate fold changes, several transcripts, including *Gm10800*, *Gm12722*, *Cyp7b1*, *Gm11961*, and *Pcdh9*, showed the largest differences between groups (Fig. 4F). The top 10 upregulated and downregulated DEGs are summarized in Fig. 4G.

GO ORA of the DEGs revealed significant enrichment of biological processes related to mitochondrial function and cellular metabolism (Fig. 4H). Downregulated transcripts were strongly associated with electron transport chain activity, NADH dehydrogenase complex assembly, oxidative phosphorylation, and other mitochondrial metabolic pathways. Additional enriched processes included organic acid transport, detoxification, and responses to xenobiotics, indicating broad effects on hepatic metabolic capacity. GO enrichment of cellular components further highlighted suppression of genes located within mitochondrial membranes, respiratory chain complexes, and cytoplasmic regions (Fig. 4I). Molecular-function categories were similarly enriched for oxidoreductase activity, amino acid and organic ion transmembrane transporter activity, and NADH dehydrogenase activity, consistent with impaired mitochondrial function (Fig. 4J).

GSEA corroborated these patterns, showing coordinated downregulation of pathways involved in mitochondrial respiration, respiratory chain complex assembly, oxidative phosphorylation, and ATP generation (Fig. 4K). Activated gene sets were comparatively limited but included processes related to cytoskeletal organization and certain cellular responses to stimuli. Hierarchical clustering of GSEA-enriched terms emphasized the coherence of the mitochondrial and metabolic signatures, grouping suppressed pathways involved in energy production, electron transport, and mitochondrial organization (Fig. 4L).

Together, these data indicate that preconception phthalate exposure produces subtle but persistent transcriptional alterations in maternal liver months after exposure has ended. Despite the modest number of DEGs, the consistent enrichment of mitochondrial and metabolic pathways across analyses suggests long-term impacts on hepatic energy homeostasis and oxidative metabolism, which may influence maternal physiological recovery following pregnancy.

### Offspring bodyweight, placental weight, and placental histology

We next evaluated the impact of phthalate exposure on pregnancy outcomes by assessing changes in fetal and placental development at E14.5. One litter from the phthalate-exposed group was removed from the analysis due to having an abnormally large litter size (21 pups) leading to overcrowding in the uterus. Exposure had no significant effect on the number of resorptions, litter size, or the distribution of pups between the left and right uterine horns at E14.5 (Supplemental Data 1, Table S8) (63). To evaluate the effects of preconception phthalate exposure on fetal growth, placental growth, and fetal:placental weight ratio, we fit a linear mixed-effects model with dose (control vs phthalate) and sex (female vs male) as fixed effects and litter included as a random intercept to account for the non-independence of fetuses within the same pregnancy.

**Figure 5:**
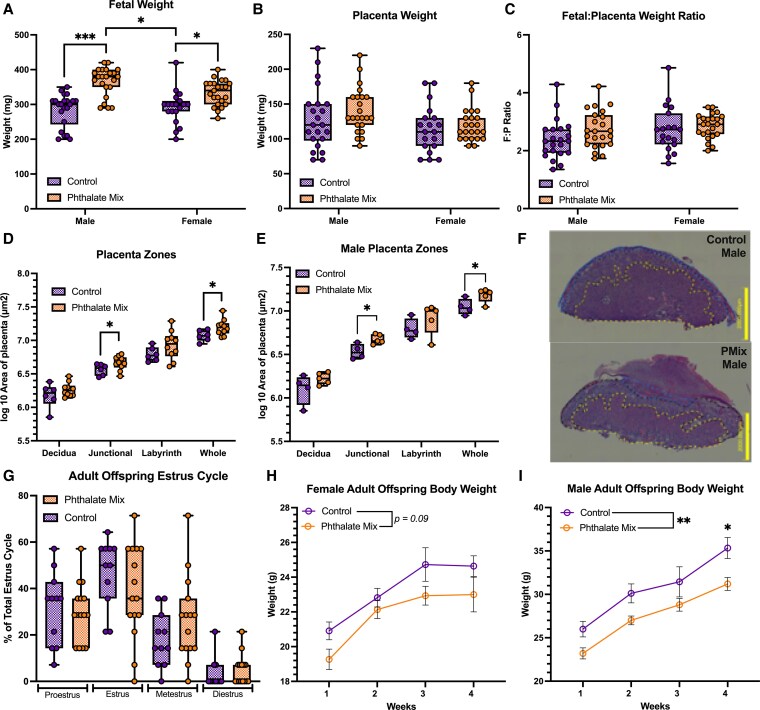
Preconception phthalate exposure significantly influenced offspring body weight during both gestation and adulthood and exerted sex-specific effects on placental morphology, most notably an increase in the male junctional zone. (A) Exposure significantly increased fetal body weight with phthalate-mixture males weighed significantly more than both control males and phthalate-mixture females but had no effect on (B) placental weight between both sexes or (C) fetal-to-placental (F:P) weight ratio (an estimate of placental efficiency); n = 19 control female fetuses; n = 22 control male fetuses; n = 23 phthalate mixture male fetuses, n = 23 phthalate mixture female fetuses. (D) A significant effect of exposure on placental areas was observed in whole placenta and junctional zone with (E) male offspring of exposed dams had a significantly larger junctional zone and whole placenta area; *n* = 2 female control; *n* = 4 male control; *n* = 5 per sex per exposed group. (F) Representative male placental histology from control and exposed groups. (G) Adult offspring estrus cycle from PND30-45 showed no significant difference between control and phthalate mixture group. Adult offspring (H) female body weight did not significantly differ between treatment groups, but (I) male offspring of exposed dams had significantly lower body weight; *n* = 11 female control; *n* = 9 male control; *n* = 15 female phthalate mixture; and n = 15 male phthalate mixture. Boxplots depict mean, min, and max. Line graphs depict mean ± SEM. Line graphs depict mean ± SEM.; *P ≤ .05; **P ≤ .01.

For fetal weight a significant main effect of dose, with phthalate-mixture exposed fetuses weighing more than controls was observed (β = + 51.05, 95% CI = 8.43-93.67, *P* = .019; Supplemental Data 1, Table S9) (63). There was no significant main effect of sex on fetal weight across doses (β = −4.55, 95% CI = −26.84-17.74, *P* = .689). However, we detected a significant dose × sex interaction (β = + 31.11, 95% CI = 0.40-61.82, *P* = .047), indicating that the effect of phthalate exposure differed between males and females. Relative to control females (290.7 mg), fetal weight increased by approximately 51 mg in phthalate-mixture females (341.8 mg) and by approximately 82 mg in phthalate-mixture males (373.3 mg) compared to control males. Post hoc pairwise comparisons confirmed that phthalate mixture significantly increased fetal weight in phthalate-mixture females compared with control females (*P* = .019, η^2^ = 0.063; Fig. 5A). Phthalate-mixture males weighed significantly more than both control males (*P* = .0001, η^2^ = 0.165) and phthalate-mixture females (*P* = .014, η^2^ = 0.148), while no difference was observed between control males and control females (*P* = .689; Fig. 5A). Together, these findings indicate that preconception phthalate exposure results in increased fetal weight, with males exhibiting a more pronounced growth response than females, and these effects remain robust after accounting for litter-level variability.

Placental weight did not differ significantly by dose (β = +12.01, 95% CI = −20.50-44.53, *P* = .469), and the main effect of sex indicated only a nonsignificant trend toward heavier placentas in males (β = +16.12, 95% CI = −2.63-34.86, *P* = .092). The dose × sex interaction was not significant (β = +12.43 mg, 95% CI = −28.59 to +23.22 mg, *P* = .570), indicating that exposure to the phthalate mixture did not alter placental weight differentially between males and females. Model-estimated marginal means reflected these findings, with placental weight ranging from 113.95 to 139.39 mg across the four sex-dose groups. Post hoc pairwise comparisons confirmed that no contrasts reached statistical significance, including the comparison between control females and control males (*P* = .130), and the comparison between phthalate-mixture females and phthalate-mixture males (*P* = .161; Fig. 5B). Collectively, these results demonstrate that preconception phthalate exposure does not significantly alter placenta weight at E14.5, and that any apparent sex differences were modest and not statistically supported after accounting for litter-level variability.

We next evaluated whether exposure to the phthalate mixture or fetal sex influenced the fetal:placental (F:P) weight ratio, a metric reflecting placental efficiency. Using the same mixed-effects model structure, we detected no significant main effect of dose (β = +.087, 95% CI = −0.44 -0.62, *P* = .748) and no significant dose × sex interaction (β = +.301, 95% CI = −0.21-0.82, *P* = .251; Fig. 5C). The main effect of sex approached but did not reach statistical significance (β = −.335, 95% CI = −0.708-0.038, *P* = .078), suggesting a possible trend toward lower F:P ratios in males compared with females. Estimated marginal means ranged from 2.127 to 2.549 across groups, with females exhibiting slightly higher ratios on average. Post hoc pairwise contrasts confirmed the absence of significant differences between any groups, including the comparison between control females and control males (*P* = .111) and between phthalate-mixture females and phthalate-mixture males (*P* = .852). Overall, these results indicate that preconception phthalate exposure does not significantly alter fetoplacental efficiency at E14.5, and that observed sex differences represent nonsignificant trends rather than statistically supported effects.

To evaluate the impact of preconception phthalate exposure on placental architecture, we used linear mixed-effects models with dose (control vs phthalate mixture) as a fixed-effect and litter as a random intercept to account for non-independence among conceptuses within litters. Analyses were performed for each placental compartment, decidua, junctional zone, labyrinth zone, and whole placenta, using log-transformed cross-sectional area as the outcome variable (Supplemental Data 1, Table S10) (63).

#### Combined-sex analyses

Across all conceptuses, phthalate exposure was associated with significant increases in junctional zone and whole placental area, whereas no dose-dependent differences were observed in the decidua or labyrinth zone (Fig. 5D). In the junctional zone, phthalate exposure resulted in a +0.102 log-unit increase in area (z = 2.05, *P* = .041; marginal R² = 0.071; conditional R² = 0.983). A similar effect was observed for the whole placenta, where phthalate exposure increased area by +0.098 log-units (z = 2.16, *P* = .031; marginal R² = 0.066; conditional R² = 0.788). In contrast, there was no significant effect of dose in the decidua (estimate = +0.020, *P* = .602) or labyrinth zone (estimate = +0.045, *P* = .271). Across all compartments, conditional R² values were high (0.69-0.98), indicating substantial variance attributable to litter, whereas marginal R² values were modest, consistent with a biologically meaningful but spatially localized phthalate effect.

#### Male-specific analyses

Because prior work suggests sex differences in placental responses to environmental exposures, we next examined male conceptuses only. The male-specific models revealed stronger and more spatially restricted phthalate effects, with significant increases in junctional zone and whole placental area but not in decidua or labyrinth (Fig. 5E).

Among male placentas, phthalate exposure increased junctional zone area by +0.156 log-units (z = 3.33, *P* = .0009; marginal R² = 0.120; conditional R² = 0.982) and increased whole placental area by +0.135 log-units (z = 3.23, *P* = .001; marginal R² = 0.119; conditional R² = 0.772). Neither the decidua (estimate = +0.027, *P* = .500) nor labyrinth (estimate = +0.041, *P* = .295) demonstrated significant dose effects in males. These compartment-specific effects indicate that male placentas are selectively sensitive to preconception phthalate exposure, particularly within the endocrine-active junctional zone.

Estrus cycle and bodyweight were also evaluated for adult offspring (PND 30-45; Supplemental Data 1, Tables S11–S13) (63). No significant effect of exposure was observed for adult female offspring estrus cycle (Fig. 5G). However, maternal phthalate exposure significantly altered offspring body weight. While this exposure had no significant impact on adult female offspring bodyweight (F (1, 24) = 2.970*, P* = .09, η^2^ = 0.11; Fig. 5H), a significant effect of phthalate exposure on adult male offspring bodyweight was observed (F (1, 22) = 8.243, *P* = .009, η^2^ = 0.27; Fig. 5I).

### Placenta—DEGs, GO, KEGG, and GSEA pathways identified in the E 14.5 placenta

PCA showed little separation between male and female control and exposed placentas (Fig. S3B) (63). However, a heatmap of all the DEGs showed clear separation via hierarchical clustering of control and exposed placentas (Fig. S3C) (63). Differential gene expression analysis (contrast matrix exposed female-control female or exposed male-control male; *P-*value ≤ .05; log2FoldChange ≥ 0) revealed significant changes in individual gene expression (*P*-adj ≤ .05; Supplemental Data 1, Tables S14 and S15) (63). A total of 518 DEGs were identified in female placenta, including 472 downregulated and 46 upregulated genes (Table S9) (63). However, in male placenta exposure only induced a significant upregulation in 9 genes. Collectively these findings suggest that exposure had a greater impact on the female placental transcriptome compared to males.

#### Female placenta

**Figure 6:**
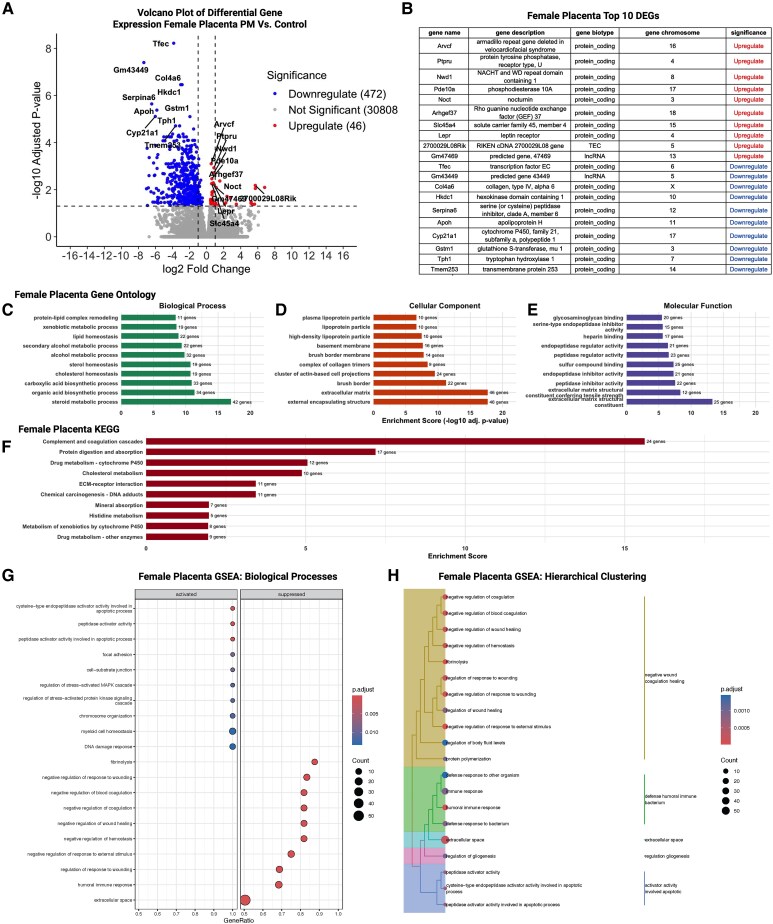
Preconception phthalate exposure markedly altered the female placental transcriptome, with differential expression of genes involved in steroid metabolism, placenta metabolic activity, adaptability, and homeostasis; n = 4 control females; n = 5 phthalate mixture females. (A) Volcano plot demonstrating upregulation in 472 genes and downregulation in 46 genes following preconception exposure to phthalate mix. (B) List of top 10 genes upregulated and downregulated. (C) GO analysis on biological processes suggested changes in placenta metabolic activity with enrichment observed including steroid metabolic processes and protein–lipid complex remodeling. (D) GO enrichment of cellular components were enriched in extracellular matrix (ECM) organization and cell–matrix interactions. (E) GO enrichment of molecular function emphasized on enzymatic detoxification, hormone or cytokine responsiveness placental metabolic adaptability. (F) KEGG pathway analysis noted alternations in drug metabolism, protein digestion and absorption, and complement and coagulation cascades. (G) Activation of cytokine-mediated and peptide hormone secretion; and suppression of ECM structural organization, cytoskeleton and cell–substrate junction noted performing GSEA in female placenta. (H) Hierarchical clustering of GSEA-enriched terms demonstrated coordinated modules encompassing immune signaling, hemostatic regulation, ECM remodeling, lipid and glucose metabolic processes, and hormone/peptide response pathways.

In the female placenta, majority of the significant DEGs were downregulated (Fig. 6A). Notable genes among the top 10 upregulated and downregulated genes include *Slc45a4* (sugar transporter), *Lepr* (regulates growth and nutrient transport), *Cyp21a1* (synthesis of steroid hormones), and *Tph1* (biosynthesis of serotonin; Fig. 6B). To characterize the molecular pathways altered by preconception phthalate exposure in female placentas at E14.5, we performed GO, KEGG, and GSEA on the DEGs identified in females (Fig. 6A and 6B).

GO biological process analysis (Fig. 6C) highlighted enrichment of metabolic pathways including protein–lipid complex remodeling, carboxylic acid and alcohol metabolic processes, secondary alcohol biosynthesis, and steroid metabolic processes. These findings suggest shifts in placental metabolic activity, particularly in pathways involved in lipid handling, oxidative metabolism, and biosynthetic functions tied to trophoblast endocrine regulation. GO cellular-component terms (Fig. 6D) were enriched for collagen-containing extracellular matrix (ECM), basement membrane structures, cortical cytoskeleton components, and basal cell surfaces. These results point toward potential alterations in ECM organization and cell–matrix interactions, processes critical for proper placental structure and nutrient exchange. GO molecular-function enrichment (Fig. 6E) identified oxidoreductase activities, peptidase and hydrolase functions, receptor binding, and diverse ligand-binding activities. Many of these functions relate to enzymatic detoxification, hormone or cytokine responsiveness, and placental metabolic adaptability.

KEGG pathway enrichment (Fig. 6F) revealed significant over-representation of processes related to the complement and coagulation cascades, protein digestion and absorption, cholesterol metabolism, drug metabolism, and chemical carcinogenesis–DNA adduct formation. Several of these pathways are involved in inflammatory signaling, vascular remodeling, and xenobiotic processing, functions that are essential for supporting fetal growth and maintaining placental homeostasis.

GSEA of biological processes (Fig. 6G) revealed activation of cytokine-mediated pathways, peptide hormone secretion, complement activation, and immune-regulatory processes. Conversely, suppressed pathways included ECM structural organization, cytoskeleton and cell–substrate junction assembly, glycosylation, and several metabolic processes. Hierarchical clustering of GSEA terms (Fig. 6H) identified coordinated modules encompassing immune signaling, hemostatic regulation, ECM remodeling, lipid and glucose metabolic processes, and hormone/peptide response pathways.

Together, these pathway-level analyses indicate that preconception phthalate exposure influences multiple aspects of female placental biology, particularly immune regulation, ECM structure, and metabolic signaling. Although the overall magnitude of transcriptional changes was moderate, the consistency across GO, KEGG, and GSEA analyses indicates coherent biological responses that may reflect subtle but coordinated placental adaptations to preconception exposure.

#### Male placenta

**Figure 7:**
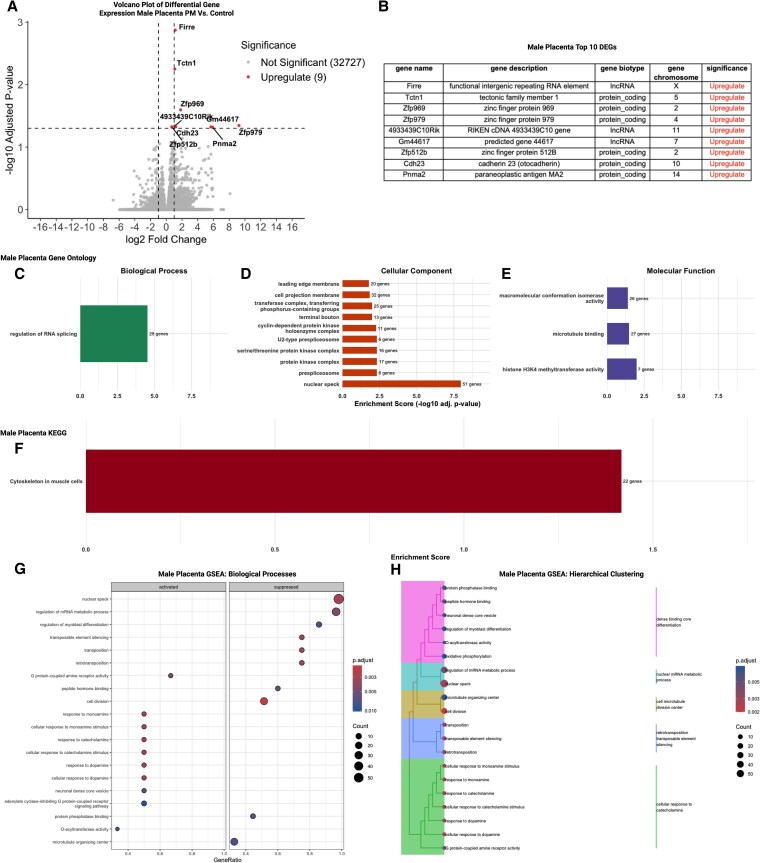
Preconception phthalate exposure produced modest transcriptomic changes in the male placenta, primarily affecting genes associated with RNA processing; n = 4 control males; n = 5 phthalate mixture males. (A) Upregulation in 9 genes following dams’ exposure to phthalate mix. (B) List of all overexpressed genes. (C) GO analysis on biological processes suggested changes only in RNA splicing. (D) Only one pathway noted in KEGG analysis. (D) GO enrichment of cellular components were enriched in RNA processing and nuclear organization like nuclear speck and spliceosomal complex. (E) GO enrichment of molecular function emphasized on macromolecular conformational isomerase activity, microtubule binding, and histone H3K4 methyltransferase activity. (F) KEGG pathway analysis noted cytoskeleton in muscle cells. GSEA analysis concluded. (G) Activation RNA splicing, RNA localization, peptide hormone signaling, and response suppressions in mitochondrial electron transport, oxidative phosphorylation, and amino acid metabolism. (H) Hierarchical clustering of GSEA-enriched terms, pathways including functional modules involving RNA processing, intracellular signaling, mitochondrial metabolism, and GPCR-mediated hormone response were observed.

In the male placenta, all 9 significant DEGs were upregulated (Fig. 7A). Majority of these genes encode for biological molecules involved in gene regulation, including long non-coding RNAs (lncRNA) and transcription factors (Fig. 7B). To evaluate whether preconception phthalate exposure altered coordinated biological pathways in male placentas at E14.5, we performed GO, KEGG, and GSEA analyses on DEGs with unadjusted *P* < .05. Consistent with the very limited number of DEGs identified in males (9 upregulated DEGs; Fig. 7A and 7B), ORA yielded a narrow set of enriched terms.

GO biological process enrichment identified only one significantly over-represented category, regulation of RNA splicing (Fig. 7C). This pathway was driven by several upregulated genes involved in RNA-binding and spliceosome-related functions, including *Firre*, *Tcf21*, and *Zfp969*, suggesting potential shifts in co-transcriptional processing despite the absence of broader transcriptional remodeling. Among cellular-component terms (Fig. 7D), enrichment was detected for structures associated with RNA processing and nuclear organization, including nuclear speck, spliceosomal complex, cyclin-dependent protein kinase holoenzyme complex, and related subnuclear domains. These terms are consistent with upregulation of spliceosome-adjacent lncRNAs and RNA-binding proteins. Molecular-function enrichment identified only a few modestly enriched categories (Fig. 7E), including macromolecular conformational isomerase activity, microtubule binding, and histone 3 lysine 4 (H3K4) methyltransferase activity, though these were supported by small gene counts and should be interpreted cautiously.

KEGG analysis yielded a single significantly enriched pathway, cytoskeleton in muscle cells (Fig. 7F), reflecting limited pathway-level signal and likely driven by a small subset of cytoskeletal or contractility-related transcripts.

In contrast to ORA methods, GSEA, which evaluates ranked gene lists independent of DEG thresholds, provided broader insight into coordinated pathway shifts (Fig. 7G). Activated processes included RNA splicing, RNA localization, peptide hormone signaling, and responses to monoamines and catecholamines. Suppressed processes were enriched for mitochondrial electron transport, oxidative phosphorylation, amino acid metabolism, and several cell-cycle and chromatin-related pathways. These patterns align with the idea that subtle regulatory or metabolic adjustments may occur even in the absence of widespread DEGs. Hierarchical clustering of GSEA biological processes (Fig. 7H) grouped enriched pathways into functional modules involving RNA processing, intracellular signaling, mitochondrial metabolism, and GPCR-mediated hormone response. Although male placentas exhibited minimal transcript-level alterations, phthalate exposure may influence post-transcriptional regulation and mitochondrial processes detectable only through gene set–based analyses.

### Liver and gonads—DEGs, GO, KEGG, and GSEA overview in PND60 liver and gonads

PCA clustering showed clear separation between the male and female liver transcriptome (Fig. S4B) (63). Furthermore, hierarchical clustering suggests that preconception phthalate exposure feminized offspring male livers and masculinized female livers with control females clustering more closely with exposed males and exposed females clustering with control males (Fig. S4C) (63). Further support for this liver feminization was observed when comparing commonly expressed genes between the four dose × sex groups. We observed greater overlap between exposed males and control females (1692 genes) than exposed males and control males (377 genes; Fig. S4D) (63). Finally, Pearson correlation analysis revealed more similar gene expression profiles between exposed female and control male (0.912) and exposed male and control female (0.913) compared to control males and control females (0.899), which may suggest exposure induced masculinization or feminization of the adult offspring liver transcriptome (Fig. S4E) (63). A total of 175 DEGs were identified in the female liver and 95 in the male liver (Tables S16 and S17) (63).

#### Female liver

**Figure 8:**
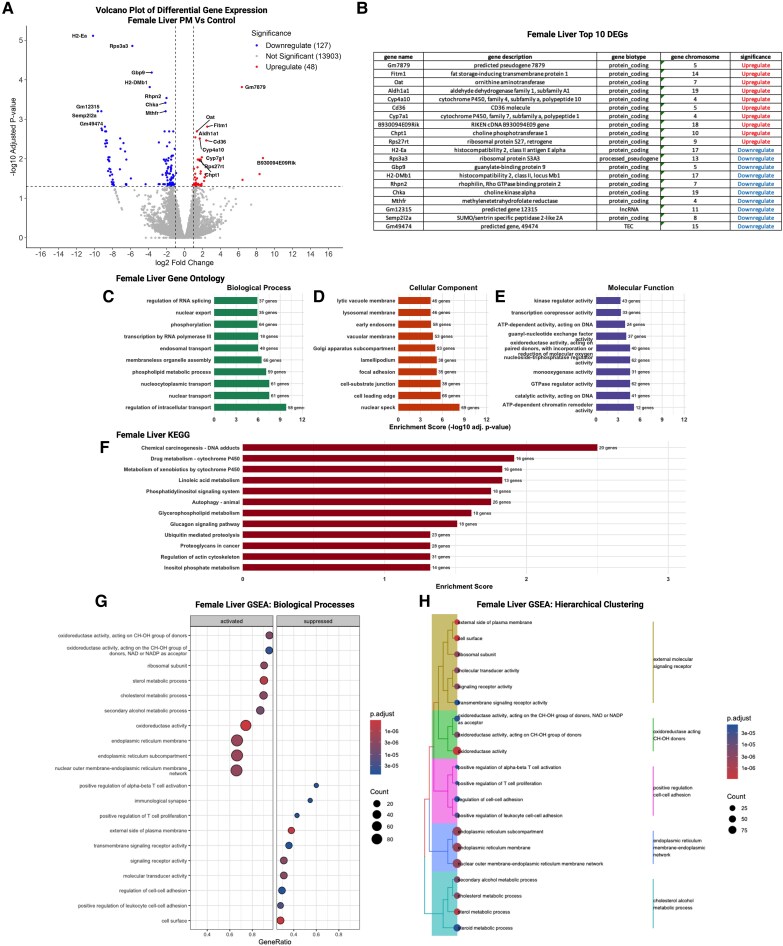
Significant transcriptomic alterations were observed in the female adult offspring liver following preconception phthalate exposure, with influencing multiple aspects of hepatic function including lipid metabolism, signaling, and transcriptional regulation; n = 3 control female; n = 3 phthalate mixture female. (A) Upregulation in 48 and downregulation in 127 following dams’ exposure to phthalate mix in female adult offspring. (B) List of top 10 genes upregulated and downregulated. (C) GO biological processes enrichment analysis suggested changes in RNA splicing and processing, phosphorylation, transcriptional regulation, and phosphatidylinositol metabolism. (D) GO cellular component enrichment revealed change in lysosomal membranes, endosomal membranes, Golgi-associated vesicles, and cytoskeletal structures. (E) GO molecular function enrichment included kinase regulator activity, transcription coactivator activity, and ATP-dependent chromatin remodeling activities. (F) Enriched terms in KEGG pathway analysis included phosphatidylinositol signaling, glycerophospholipid metabolism, glycogen metabolism. (G) GSEA demonstrated activation of pathways related to steroid metabolism, cholesterol biosynthesis, and metabolic remodeling, and suppression of pathways linked to immune regulation, inflammatory signaling, and glucose metabolic processes. (H) Responses were grouped in lipid metabolism, steroid biosynthetic activity, extracellular signaling, and transcriptional regulation performing hierarchical clustering.

Similar to the placenta, significant DEGs in adult female offspring livers were predominantly downregulated (Fig. 8A). Among the top DEGs were regulators of lipid metabolism (eg, *Cyp4a10*, *Cyp7a1*), folate and one-carbon metabolism (*Mthfr*), antigen presentation (*H2-DMB1*), oxidative stress response (*Gstm2*), and xenobiotic processing (*Cyp2b9*, *Cyp2b10*) (Fig. 8B). GO biological process enrichment revealed significant over-representation of pathways linked to RNA splicing and processing, phosphorylation, transcriptional regulation, and phosphatidylinositol metabolism (Fig. 8C). These categories suggest coordinated modulation of nuclear regulatory processes and intracellular signaling pathways in exposed females. Enrichment of cellular-component terms (Fig. 8D) highlighted lysosomal membranes, endosomal membranes, Golgi-associated vesicles, and cytoskeletal structures, indicating potential disruptions in vesicular trafficking, organelle dynamics, and intracellular transport. Molecular-function enrichment (Fig. 8E) included kinase regulator activity, transcription coactivator activity, and ATP-dependent chromatin remodeling activities, consistent with altered transcriptional and post-transcriptional control mechanisms following exposure.

KEGG pathway analysis identified significant enrichment in phosphatidylinositol signaling, glycerophospholipid metabolism, glycogen metabolism, and pathways involved in chemical carcinogenesis and cytochrome P450-mediated drug metabolism (Fig. 8F). These findings align with known roles of the liver in lipid processing, energy storage, and xenobiotic detoxification.

Finally, GSEA revealed coordinated activation of pathways related to steroid metabolism, cholesterol biosynthesis, and metabolic remodeling, alongside suppression of pathways linked to immune regulation, inflammatory signaling, and glucose metabolic processes (Fig. 8G). Hierarchical clustering (Fig. 8H) grouped these responses into functional modules representing lipid metabolism, steroid biosynthetic activity, extracellular signaling, and transcriptional regulation. Collectively, these enrichment patterns suggest that preconception phthalate exposure influences multiple aspects of hepatic function in females, particularly lipid metabolism, phosphoinositide signaling, and transcriptional regulation.

#### Male liver

**Figure 9:**
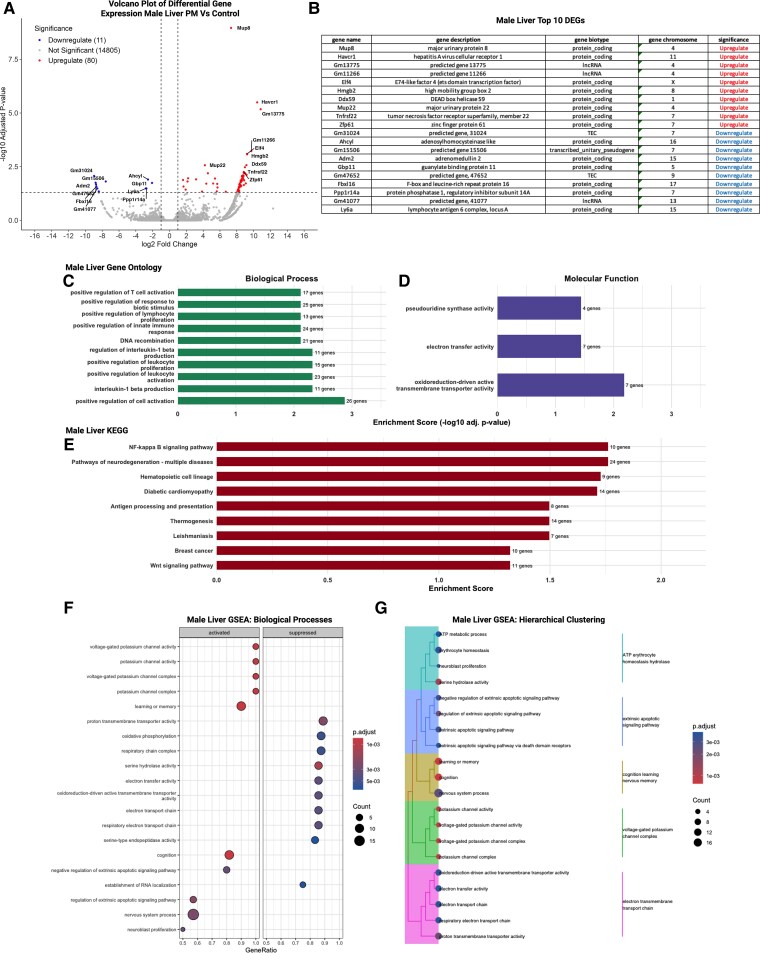
Transcriptomic alterations were observed in the male adult offspring liver following preconception phthalate exposure, with influencing multiple aspects of inflammatory responses and signaling processes; n = 2 control male; n = 3 phthalate mixture male. (A) Upregulation in 80 and downregulation in 11 following dams’ exposure to phthalate mix in male adult offspring. (B) List of top 10 genes upregulated and downregulated. (C) GO biological processes enrichment analysis suggested changes in T-cell activation, lymphocyte differentiation, interleukin-1β production, and regulation of inflammatory signaling. (D) GO molecular-function enrichment included pseudouridine synthase activity, oxidoreductase activity, and electron-transfer functions among the top categories. (E) Enriched terms in KEGG pathway analysis included including NF-κB signaling, hematopoietic cell lineage, Wnt signaling, thermogenesis, and antigen processing and presentation. (F) GSEA demonstrated activation of pathways related to potassium-channel activity, oxidative phosphorylation, electron transport chain function, and protein transmembrane transport, alongside suppression of pathways associated with apoptotic signaling and receptor-mediated processes. (G) Responses were grouped in mitochondrial metabolism, ion-channel regulation, immune response, and apoptotic signaling performing hierarchical clustering.

In the male liver, DEGs were predominantly upregulated, with 80 upregulated and 11 downregulated transcripts identified (Fig. 9A and 9B). Among the top DEGs were genes involved in immune signaling (eg, *Elf4*, *Tnfrsf22*), stress response (*Herc1*), peptide hormone signaling (*Adm2*), and immune modulation (*Ly6a*) (Fig. 9B). GO enrichment analysis revealed significant over-representation of biological processes related to T-cell activation, lymphocyte differentiation, interleukin-1β production, and regulation of inflammatory signaling (Fig. 9C). Molecular-function enrichment identified pseudouridine synthase activity, oxidoreductase activity, and electron-transfer functions among the top categories (Fig. 9D). In contrast to female liver, KEGG analysis yielded multiple enriched pathways, including NF-κB signaling, hematopoietic cell lineage, Wnt signaling, thermogenesis, and antigen processing and presentation (Fig. 9E). GSEA further highlighted coordinated activation of pathways related to potassium-channel activity, oxidative phosphorylation, electron transport chain function, and protein transmembrane transport, alongside suppression of pathways associated with apoptotic signaling and receptor-mediated processes (Fig. 9F). Hierarchical clustering of GSEA terms grouped these into modules associated with mitochondrial metabolism, ion-channel regulation, immune response, and apoptotic signaling (Fig. 9G). Collectively, these enrichment patterns suggest that preconception phthalate exposure influences multiple aspects of inflammatory responses and intracellular signaling processes in male livers.

#### Female ovaries

PCA did not show consistent separation of control and exposed ovaries, however hierarchical clustering did reveal a dose-dependent separation (Fig. S5) (63) To determine whether preconception phthalate mixture exposure produced lasting molecular effects in the adult female reproductive system, we performed RNA-seq on PND60 ovaries from offspring of control and phthalate-exposed dams (Supplemental Data 1, Table S18) (63). Overall, transcriptomic responses in the ovary were minimal. Differential expression analyses revealed only two significant DEGs, *Samt1c* and *Gm33450*.

Consistent with the limited number of DEGs, GO molecular-function enrichment identified only small clusters of over-represented categories, primarily involving cytokine receptor binding, voltage-gated ion-channel activity, and chemokine activity (Fig. S6A) (63). These enrichments were driven by a small subset of genes showing trends toward differential expression but did not reflect broad transcriptional remodeling.

At the pathway level, KEGG enrichment showed over-representation of pathways related to cytokine receptor Interactions, chemokine signaling, and steroid biosynthesis (Fig. S6B) (63). These pathways were similarly derived from small gene sets and should be interpreted cautiously given the limited DEG count.

GSEA analysis identified modest enrichment of biological processes related to immune signaling regulation, response to bacterial or biotic stimuli, and signal transduction, with both positively and negatively enriched pathways represented (Fig. S6C) (63). Hierarchical clustering of enriched processes (Fig. S6D) (63) organized these responses into functional modules involving adaptive immune signaling, cellular response to external stimuli, and chemotaxis, although these patterns again reflected broader trends rather than robust transcript-level shifts. Taken together, these results indicate that preconception phthalate exposure produced minimal lasting transcriptional effects in the adult female ovary, with only two DEGs identified and modest enrichment of immune- and signaling-related pathways.

#### Male testes

To evaluate whether preconception phthalate exposure produced long-term transcriptional effects in male offspring gonads, we performed differential expression analysis on PND60 testicular tissue (Supplemental Data 1, Table S19) (63). Similar to the female ovary, PCA did not show consistent separation of control and exposed ovaries, however hierarchical clustering did reveal a dose-dependent separation (Fig. S7) (63) Overall, the male gonad transcriptome was minimally affected. Only a single gene, *Rps2-ps13* (a ribosomal protein pseudogene), met significance thresholds. No additional genes approached statistical significance, and volcano plots showed a uniformly narrow distribution of fold changes across the transcriptome, indicating an absence of broad transcriptional perturbation.

Because only a single gene (Rps2-ps13) met the threshold for differential expression in PND60 male gonads, GO and KEGG ORA did not identify any significantly enriched pathways. However, GSEA, identified several pathways with nominal adjusted *P*-values < .05. These enriched terms reflected coordinated but small magnitude shifts in genes involved in immune signaling, cellular responses to external stimuli, and metabolic processes (Fig. S8A and S8B) (63). Given the extremely limited number of DEGs, these GSEA findings should be interpreted cautiously as exploratory and hypothesis-generating rather than indicative of robust transcriptional reprograming.

Together, these analyses indicate that preconception exposure did not measurably alter gene expression programs in male testes, in contrast to the more pronounced transcriptional sensitivity observed in placenta and liver tissues. This absence of effect may reflect tissue-specific resilience, compensatory mechanisms during development, or insufficient exposure intensity to perturb long-term testicular molecular function.

## Discussion

EDCs, including phthalates, are pervasive in the environment and are found in a wide array of consumer products such as plastics, cosmetics, and personal care items (82, 83). Women of reproductive age are especially vulnerable to these exposures, which have been increasingly linked to reproductive dysfunction and adverse pregnancy outcomes (60, 62). Although gestational exposure to phthalates has been extensively studied, emerging evidence suggests that exposures prior to conception may also disrupt critical early reproductive events and fetal programing (84-86). However, the preconception window remains a poorly understood yet potentially sensitive period. This pilot study provides new evidence that preconception exposure to a human-relevant phthalate mixture disrupts maternal endocrine function, alters placental structure and gene expression, and impairs offspring development in a sex-specific manner. By modeling environmentally relevant exposure during the preconception window, a critical yet understudied period, our findings expand current understanding of when and how EDCs may impact reproductive and developmental health.

### Preconception exposure may alter maternal endocrine function

Consistent with prior studies examining phthalate-induced reproductive disruption, this preliminary investigation identified trends toward altered estrus cyclicity following 30-day preconception exposure, including a tendency toward prolonged proestrus and shortened metestrus stages (87-90). Although no statistically significant changes in maternal body weight or fertility outcomes (implantation or litter size) were detected, these cyclicity patterns raise the possibility of subtle endocrine effects on the hypothalamic–pituitary–gonadal axis (91). Importantly, these observations occurred in the absence of overt maternal toxicity, suggesting that any endocrine impacts are likely modest and require confirmation in larger cohorts. Because estrus cyclicity influences the timing and hormonal environment of conception, even minor perturbations could have downstream implications for placental development and fetal health, though such effects remain speculative based on the current dataset (92-95). The limited sample size in this study may have hindered our ability to detect subtle or moderate treatment-related differences. Future investigations into maternal fertility endpoints related to preconception phthalate exposure should prioritize increasing the sample size and rounding out the fertility endpoints to include time to pregnancy, mating success, and gestational length.

### Maternal hepatic metabolism appears sensitive to preconception phthalate exposure

The maternal liver transcriptomic data revealed that preconception phthalate exposure produces a modest yet biologically coherent pattern of transcriptional change that persists over a month after exposure cessation and postpartum physiological recovery. Although the number of DEGs was relatively small, multiple independent enrichment approaches, GO over-representation, KEGG analysis, GSEA, and hierarchical clustering, converged on a shared signature marked by downregulation of oxidative phosphorylation, mitochondrial electron transport, and associated metabolic pathways. This recurrent suppression of mitochondrial programs suggests that phthalate exposure may produce lingering impacts on hepatic energy metabolism, even when overt maternal toxicity is absent and systemic indicators such as body weight remain unchanged.

The enriched biological processes identified through GO analysis highlight disruptions in organic acid metabolism, detoxification pathways, and responses to xenobiotics and oxidative stress. These functional categories align with the liver's central role in biotransformation and raise the possibility that preconception exposures alter hepatic readiness to manage metabolic or toxicological challenges later in life (96, 97). KEGG pathway enrichment further reinforced this interpretation, identifying changes within oxidative phosphorylation, amino acid transport, and membrane-associated metabolic hubs. Together, these findings are consistent with prior evidence that phthalates can impair mitochondrial function, alter lipid handling, and promote oxidative stress in hepatic and extrahepatic tissues (98-101).

GSEA provided additional resolution by revealing coordinated suppression of mitochondrial respiratory chain complexes, NADH dehydrogenase activity, and ATP-linked metabolic processes. Notably, only a small number of pathways showed positive enrichment, largely related to intracellular transport and cytoskeletal remodeling, responses that may reflect compensatory structural adjustments rather than primary targets of phthalate toxicity. Hierarchical clustering of enriched terms demonstrated that mitochondrial and metabolic pathways formed the most densely interconnected modules, suggesting that these systems may be central nodes of hepatic vulnerability in the context of preconception endocrine disruption.

Although the functional implications of these transcriptional changes remain speculative, the observed pattern is consistent with the possibility that preconception phthalate exposure subtly shifts hepatic metabolic programing. Because the liver undergoes extensive remodeling during pregnancy and lactation, even small disruptions in mitochondrial function could influence how dams recover metabolically after weaning or respond to future physiological demands (102-104). The persistence of these changes to PND 100 underscores that the window before conception may have lasting effects on maternal physiology, independent of gestational exposures.

Importantly, the magnitude of these transcriptional alterations is modest, and additional studies with larger sample sizes, metabolomic profiling, and functional assays will be necessary to determine whether these gene expression changes translate into measurable metabolic outcomes. Nonetheless, the convergence of multiple analytical approaches on mitochondrial and metabolic pathways provides a coherent biological narrative and highlights the importance of considering maternal recovery, not just fetal and placental outcomes when evaluating the long-term consequences of preconception chemical exposures.

### Preconception exposure may alter placental architecture and drive early growth shifts

While implantation rates and litter sizes did not differ between groups, we observed a significant increase in fetal body weight associated with preconception phthalate exposure, with the greatest impact observed in male fetuses at E14.5. Because placental structure is a major determinant of fetal growth, and prior studies show that phthalates can disrupt placental architecture and trophoblast development, these differences in fetal size prompted us to examine placental morphology more closely (105-108). In our exploratory histological assessment, we also noted an apparent expansion of the junctional zone in male placentas, a compartment enriched in hormone-producing spongiotrophoblasts that plays a critical role in maternal–fetal signaling and fetal growth regulation (109). Prior work demonstrates that placental structure is sex-specific under normal conditions and that male placentas often show heightened sensitivity to environmental perturbations; however, our study was not powered to formally test sex-specific differences in placental zone architecture. Even so, the pattern we observed, an enlarged junctional zone accompanied by increased fetal weight, aligns with established literature on male-biased vulnerability to endocrine disruption.

Previous studies have shown that phthalate exposure during gestation can lead to abnormal placental morphology, and our results extend these findings to the preconception period (108, 110). Because the present histological analyses were performed in a limited number of litters, these findings should be viewed as preliminary and hypothesis-generating. Larger, fully powered studies that explicitly incorporate fetal sex as a biological variable will be required to determine whether these zone-specific differences represent consistent structural alterations and to elucidate the mechanisms through which preconception exposures may influence placental organization.

### Female placentas exhibit greater transcriptional sensitivity compared to males

Our transcriptomic analyses indicate that female placentas displayed substantially greater transcriptional sensitivity to preconception phthalate exposure compared to males, consistent with well-documented fetal sex differences in placental adaptability, endocrine function, and stress responsiveness. GO, KEGG, and GSEA analyses collectively revealed coordinated disruption across metabolic, structural, immune-regulatory, and neurodevelopmentally relevant pathways, suggesting that even modest preconception exposures may alter the molecular landscape of early placental development.

#### Structural organization, ECM remodeling, and metabolic regulation

Pathway enrichment findings strongly pointed to altered processes associated with extracellular matrix (ECM) dynamics, cytoskeletal organization, and metabolic homeostasis. GO categories including extracellular structure organization, collagen-containing ECM, cell adhesion, and actin filament organization were significantly enriched in the female placenta transcriptome. These categories were driven by downregulation of several ECM and adhesion-associated genes, such as *Col4a6*, *Arvcf*, and multiple protocadherins (eg, *Pcdh9*) (111-113). KEGG enrichment further supported perturbations in focal adhesion, ECM—receptor interactions, and tight junction pathways, essential processes for proper placental morphogenesis and labyrinth expansion.

GSEA additionally highlighted coordinated downregulation of pathways related to oxidative phosphorylation, mitochondrial electron transport, and lipid metabolic processes. This aligns with suppressed expression of metabolic regulators such as *Gstm1*, a glutathione metabolism enzyme critical for reactive oxygen species detoxification (114-117). Reduced *Gstm1* expression may heighten vulnerability to oxidative stress, a known mechanism underlying phthalate-induced cellular injury. Upregulation of nocturnin (*Noct*), a circadian-regulated mediator of lipid turnover and energy utilization, suggests potential rewiring of metabolic timing and nutrient handling (118, 119). Given the placenta's reliance on highly orchestrated metabolic fluxes to support growth, these findings raise the possibility that preconception phthalate exposure produces subtle but functionally meaningful shifts in placental energy homeostasis. Taken together, enrichment across structural and metabolic pathways indicates that phthalate exposure may not simply alter isolated genes but may instead provoke broad, coordinated changes that influence placental architecture and nutrient regulation.

#### Immune modulation, inflammatory signaling, and xenobiotic response

GO and KEGG enrichment also identified disruptions in immune-associated pathways, including complement activation, coagulation cascades, cytokine signaling, and xenobiotic metabolism. These pathway-level findings were supported by differential expression of genes involved in inflammation, steroid handling, and stress signaling. One notable example is the downregulation of *Serpina6*, which encodes corticosteroid-binding globulin (CBG), a primary regulator of glucocorticoid availability. Perturbation of CBG can alter fetal exposure to maternal glucocorticoids, with downstream impacts on immune maturation and stress responsivity (120-122). Additional decreases in transcripts involved in oxidative defense (eg, *Gstm1*) and xenobiotic detoxification further suggest impaired ability of the placenta to manage chemical and inflammatory stressors (114-117).

GSEA findings reinforced this pattern by identifying negative enrichment of pathways linked to inflammatory regulation, steroid biosynthesis, and immune effector processes, indicating suppression rather than activation of key maternal–fetal signaling networks. Given the placenta's immunological role in balancing maternal tolerance with fetal protection, such alterations may have functional implications for gestational immune programing.

#### Neurodevelopmental and epigenetic programing pathways

Beyond effects on structure and immune regulation, several of the DEGs map to pathways implicated in fetal neurodevelopment and epigenetic regulation. Notably, we observed downregulation of *Tph1*, a rate-limiting enzyme in placental serotonin synthesis (123-126). Since placental serotonin contributes to forebrain development during early gestation, reductions in *Tph1* expression may influence neurodevelopmental trajectories.

Multiple lncRNAs were also altered, consistent with emerging literature showing that phthalates can reshape epigenetic landscapes by modifying chromatin accessibility, transcription factor binding, and co-transcriptional processes (123-126). KEGG and GSEA analyses highlighted enrichment in pathways associated with RNA processing, mRNA splicing, and chromatin assembly, supporting the concept that early epigenetic reprograming may be an underrecognized consequence of preconception phthalate exposure.

Although male placentas exhibited far fewer DEGs, enrichment of RNA splicing and post-transcriptional regulation pathways suggests that subtle regulatory alterations may still occur even in the absence of broad transcriptional changes. This aligns with recent findings that environmental endocrine disruptors, including bisphenol A and phthalates, can impair RNA-binding proteins and spliceosome components, producing functional consequences not immediately apparent from DEG counts alone (127-131).

Taken together, these GO, KEGG, and GSEA findings show that preconception phthalate exposure elicits coordinated transcriptional responses in female placentas across multiple functional domains. Structural remodeling, immune regulation, metabolic pathways, and neurodevelopmental signaling represent the most prominently affected systems. Importantly, the convergence of differential expression across ECM, metabolic, and inflammatory pathways suggests that phthalate exposure may subtly shift the balance of placental growth and function during a critical window of fetal development.

### Male placenta structural alterations are associated with postnatal growth outcomes

Although male placentas were comparatively less transcriptionally plastic than female placentas, they exhibited distinct structural alterations, including expansion of the junctional zone at E14.5, and these early morphological differences were accompanied by postnatal consequences. Male offspring from phthalate-exposed litters showed significantly reduced weight gain in adulthood, suggesting persistent metabolic or endocrine programing despite minimal overt transcriptional disruption in the placenta at mid-gestation. Together, these findings support the concept that male placentas may rely more heavily on post-transcriptional or epigenetic regulatory mechanisms and, as a result, may be more vulnerable to long-term physiological consequences following preconception exposure.

### Disruption of placental and hepatic pathways links preconception exposure to lifelong growth effects

Preconception phthalate exposure elicited sex-specific transcriptomic changes in the adult offspring liver. Hierarchical clustering and Pearson correlation analysis revealed a “feminization” of male livers and “masculinization” of female livers, suggesting disruption of normal sex differences in hepatic gene expression (63). In females, upregulation of metabolic genes such as *Cyp4a10* and *Cyp7a1* may indicate enhanced fatty acid oxidation and bile acid synthesis, processes that, when dysregulated, can disrupt lipid homeostasis and energy balance (132-134). While increased bile acid synthesis may initially support nutrient absorption, excessive activity could impair cholesterol regulation and contribute to altered metabolic programing (135). Concurrent downregulation of *Mthfr* points to disrupted one-carbon metabolism, with implications for epigenetic regulation and endocrine signaling (136, 137). In males, several immune- and signaling-related genes were differentially expressed, with *Elf4* and *Tnfrsf22* upregulated but *Adm2* and *Ly6a* downregulated. Loss of *Adm2*, a peptide hormone involved in vascular tone and metabolic regulation, and *Ly6a*, a modulator of immune responsiveness, suggests impaired hepatic signaling capacity and reduced adaptability to stress (138-141). These changes could compromise the balance between immune regulation and growth hormone/insulin-like growth factor pathways, constraining anabolic growth (142-144). Together, these liver disruptions provide a mechanistic link to the reduced adult bodyweight observed in both sexes, statistically significant in males and trending downward in females, through sex-specific alterations in metabolic, epigenetic, and endocrine signaling pathways. Importantly, these hepatic findings parallel the divergent transcriptomic responses observed in the placenta, underscoring the placenta–liver axis as a central mediator of growth and metabolic programing following preconception phthalate exposure.

The contrasting effects of phthalate exposure on fetal vs adult growth may reflect a classic example of developmental reprograming described by the Developmental Origins of Health and Disease (DOHaD) framework (143, 145, 146). At mid-gestation, increased fetal weight likely results from altered placental nutrient transfer and compensatory endocrine activity, consistent with the expansion of the junctional zone in male placentas and the upregulation of metabolic pathways in female placentas (147, 148). These shifts may transiently enhance nutrient delivery and promote rapid growth *in utero*. However, such adaptations can create long-term tradeoffs. Dysregulated hepatic pathways in offspring, including excessive bile acid synthesis (*Cyp7a1*), altered fatty acid metabolism (*Cyp4a10*), disrupted one-carbon metabolism (*Mthfr*), and impaired immune/endocrine signaling (*Adm2, Ly6a*), are indicative of reduced metabolic flexibility postnatally. Over time, this mismatch between early placental-driven overnutrition and later hepatic dysfunction likely contributes to impaired nutrient utilization, antagonism of growth hormone/insulin-like growth factor signaling, and ultimately the reduced adult bodyweights observed in both sexes. This trajectory parallels the “Barker hypothesis,” which posits that perturbations in the intrauterine environment can lead to a thrifty phenotype: short-term survival advantages during development that predispose offspring to long-term growth restriction and metabolic disease (149, 150).

Importantly, these mechanistic insights align with human epidemiological evidence. Several studies have reported associations between maternal phthalate exposure and altered birthweight, with outcomes ranging from fetal overgrowth to growth restriction depending on timing, dose, and mixture composition. For example, prenatal phthalate exposure has been linked to changes in placental size and function (106, 107) as well as increased risk of preterm birth (151). Longitudinal cohort studies have further demonstrated that maternal and paternal preconception exposures predict reduced birth size and altered childhood growth trajectories (52, 86). These findings parallel the growth patterns observed in our murine model, suggesting that the placenta–liver axis may represent a conserved mechanism of endocrine-metabolic reprograming across species. Collectively, this evidence highlights the translational relevance of our work and underscores the need to consider preconception exposures in assessing the intergenerational impacts of environmental chemicals on human health.

### Preconception exposure yields minimal lasting effects on offspring gonadal gene expression

Preconception phthalate exposure produced minimal long-term transcriptional alterations in the gonads of PND60 offspring. In females, only two genes (*Samt1c* and *Gm33450*) met statistical significance, and enrichment analyses revealed small, low-confidence clusters of immune- and signaling-related pathways, reflecting subtle trends rather than robust molecular remodeling. Male testes exhibited even greater transcriptional stability, with a single pseudogene (*Rps2-ps13*) identified as differentially expressed and no significant GO or KEGG enrichments detected. Although GSEA identified several nominally enriched pathways in both sexes, these patterns were modest in magnitude and should be interpreted cautiously given the limited DEG counts. Together, these findings suggest that the ovary and testis are comparatively resilient to long-term transcriptional reprograming from preconception exposures, in contrast to the more pronounced effects observed in placenta and liver. These results underscore the tissue-specific nature of phthalate sensitivity and highlight the need for future studies with larger sample sizes and functional readouts to fully evaluate reproductive consequences across developmental windows.

### Conclusions and limitations

Collectively, these exploratory findings demonstrate that preconception exposure to a human-relevant phthalate mixture disrupts maternal endocrine function, alters placental structure and transcriptome, and impairs offspring growth in a sex-specific manner. Our results reinforce the placenta's role as a sensitive and integrative target of environmental exposures, mediating both immediate and long-term effects on offspring health. Moreover, they highlight the preconception window as a critical, yet often overlooked, period of vulnerability in the context of DOHaD.

Several limitations should be acknowledged to contextualize the findings of this pilot investigation. First, we evaluated a single, environmentally relevant dose of a phthalate mixture, which constrains our ability to assess dose–response relationships or identify effect thresholds. Future studies incorporating multiple exposure levels will be needed to delineate nonlinear or low-dose effects typical of EDCs.

Second, we were not able to measure internal phthalate metabolite concentrations in maternal or fetal tissues. Because the study was designed as an exploratory assessment focused on early pregnancy, placental development, and offspring outcomes, we did not perform the repeated biological sampling (eg, serial serum or urine collection) required for toxicokinetic analyses. As a result, internal dose cannot be directly inferred. We note this constraint explicitly and highlight that future work will incorporate targeted metabolite quantification, measurement of placental and fetal burdens, and physiologically based toxicokinetic modeling to strengthen cross-species relevance and mechanistic interpretation.

Third, statistical power was limited by the number of dams included in the study. Larger sample sizes will be essential for confirming the observed trends and supporting more robust sex-stratified analyses. In parallel, several reproductive endpoints, such as time to pregnancy, mating success, and gestational length, were not measured. Although we did not detect statistically significant alterations in estrous cyclicity, trends toward prolonged proestrus and shortened metestrus suggest potential maternal endocrine disruption. Future investigations should incorporate expanded fertility metrics and maternal hormone profiling (eg, estradiol and progesterone), as well as reproductive hormone assessments in adult offspring to evaluate potential intergenerational effects.

Fourth, although CO₂ inhalation is a widely accepted and humane euthanasia method, it has the potential to induce transient hypoxia- and stress-responsive transcriptional changes in certain tissues, particularly the central nervous system (64). These effects are far less pronounced in peripheral or extraembryonic tissues, especially when collected immediately after euthanasia, as was done here. Compared to other methods of euthanasia, such as pentobarbital, isoflurane, and propofol, CO_2_ asphyxiation has been shown to have less of an impact on the expression and activity of gene and protein targets (64). Moreover, all animals were euthanized using an identical standardized protocol, meaning any nonspecific effects would contribute only to background variability and not systematic treatment differences. Nonetheless, CO₂ remains a potential source of minor gene expression variability and is noted as a study limitation.

Fifth, for placental transcriptomic analyses, sample selection was constrained by tissue quality and RNA integrity. To reduce intrauterine endocrine variability, we prioritized placentas from fetuses flanked by mixed-sex neighbors, a context known to moderate local hormonal microenvironments (69, 70), and selected those with the highest RIN values and well-preserved histoarchitecture. While this approach improved data quality, we acknowledge that we did not explicitly control for uterine position in a broader anatomical sense (eg, left vs right horn), which may become more relevant in models of uterine crowding or impaired implantation. This is noted as an additional limitation that future studies with larger cohorts can address.

Finally, although CD-1 mice serve as a valuable outbred model, species-specific differences in placental structure, steroidogenesis, and metabolic capacity may limit direct extrapolation to human pregnancy.

Despite these constraints, this study provides novel, sex-specific insights into how preconception phthalate exposure can influence placental biology and maternal and offspring health, underscoring the importance of expanding reproductive toxicology frameworks to include the preconception period as a critical and understudied window of vulnerability.

## Data Availability

Original data generated and analyzed during this study are included in this published article or in the data repositories listed in References.
